# Author Correction: Controlling invasive ant species: a theoretical strategy for efficient monitoring in the early stage of invasion

**DOI:** 10.1038/s41598-018-28018-4

**Published:** 2018-06-20

**Authors:** Shumpei Ujiyama, Kazuki Tsuji

**Affiliations:** 1 Innovation Science Course, School of Environment and Society, Tokyo Institute of Technology, 2-12-1 Ookayama, Meguro-ku, Tokyo 152-8550 Japan; 2 Department of Subtropical Agro-Environmental Sciences, University of the Ryukyus, Senbaru 1, Nishihara, Okinawa 903-0213 Japan

Correction to: *Scientific Reports* 10.1038/s41598-018-26406-4, published online 23 May 2018

The Acknowledgements section in this Article was omitted. The Acknowledgements section should read:

“This study was supported by Grants-in-Aid for Scientific Research 15H02652 and 15H04425 (K.T.) from the Japan Society for the Promotion of Science.”

