# Supplementary material for: Controlling invasive ant species: a theoretical strategy for efficient monitoring in the early stage of invasion
Source: Sci Rep. 2018 May 23;8:8033. doi: 10.1038/s41598-018-26406-4 (PMC5966387; doi:10.1038/s41598-018-26406-4)
Supplement: Supplementary file 1 — Supplementary Information [file 41598_2018_26406_MOESM1_ESM.pdf]

Supplementary Information for Controlling invasive ant species: a theoretical strategy for efficient monitoring in the early stage of invasion

Shumpei Ujiyama[1]* and Kazuki Tsuji[2]

[1]*Innovation Science Course, School of Environment and Society, Tokyo Institute of Technology, 2-12-1 Ookayama, Meguro-ku, Tokyo, 152-8550, Japan.* [2]*Department of Subtropical Agro-Environmental Sciences, University of the Ryukyus, Senbaru 1, Nishihara, Okinawa, 903-0213, Japan.*

* ujiyama.s.aa@m.titech.ac.jp

1 The focus of this research

We aim to investigate the detection rate with variation in the monitoring area and spatio-temporal intensity of monitoring. Therefore, we assume that the rate of capture of fire ants when the trap is placed in the foraging territory around the fire ant nest (here called the "trap efficacy") is one. There are two justifications for this assumption: (1) to maintain the model mathematically solvable; and (2) to focus on the issue.

(1) To maintain the model mathematically solvable

When introducing a trap efficacy p $(0 \le p \le 1)$, it is easily deduced that the observable ratio $O(t)$ will satisfy $O(t) < 1$ (see Supplementary Information (SI) section 3 for definition of the *observable ratio*). For simplicity, assume that traps are set in patterns shown in Fig. S 1.

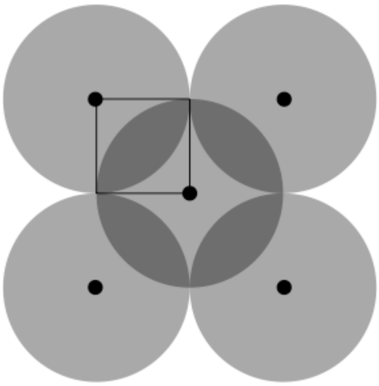

Fig. S 1 Location of traps in a thought experiment.

Black dots denote traps. Detectable areas are shown in grey, and the dark grey areas are the overlapping areas of detectable areas.

The entire monitoring area could be divided into sub-areas which all of them are equally covered by the detectable area (one of these areas is the square region surrounded by the black line in Fig. S 1).

Focusing on the square region, we can define $O(t)$ as the ratio of the area covered by the detectable area to the square area, which the area covered is schematically shown as following.

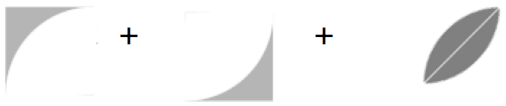

If the trap efficacy satisfies $p=1$, then the observable ratio satisfies $O(t)=1$ because the detectable area covers the entire square region. However when $p<1$, from the following schematic, it is shown that $O(t)<1$.

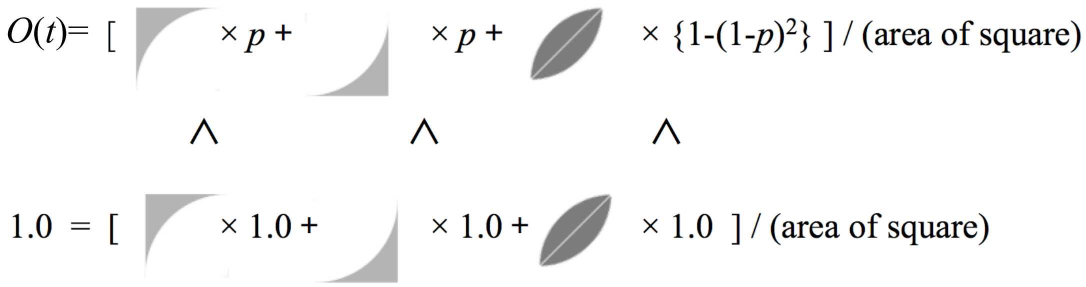

This thought experiment implies that the observable ratio (and thus detection rate) will never reach one if we incorporate such trap efficacy that is below one. The implication holds true even when considering more realistic situations such as following.

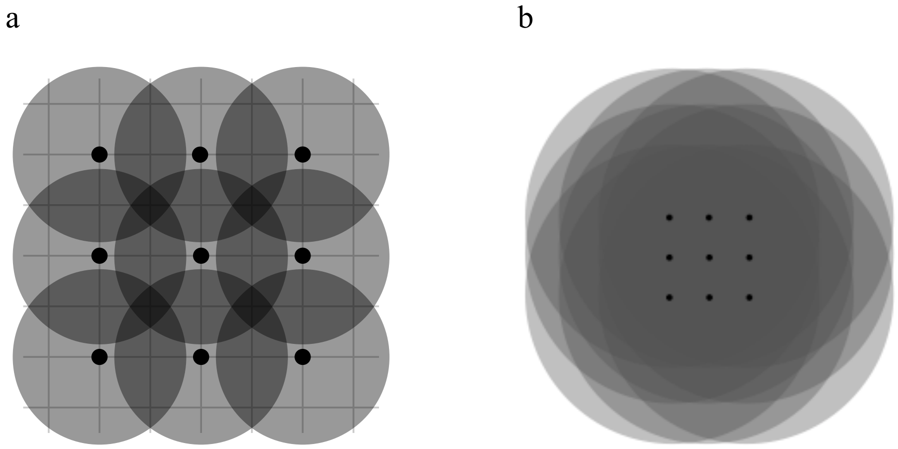

Fig. S 2 Examples of further realistic situations.
(a) Detectable areas when the radius of the detectable area is relatively small. (b) Detectable areas when the radius of the detectable area is large such that neighboring detectable areas are overlapping.

Thus if we incorporate trap efficacy, because the detection rate will never reach one, the available implication from the model would be "decrease the bait interval as less as possible to increase

detection rate", which is not an insightful implication.

Furthermore, we will not be able to formulate the detection rate if we incorporate trap efficacy. As we showed in the above discussion, it is required to formulate every overlapping and non-overlapping areas in order to formulate the observable ratio. However, this becomes extremely difficult even in a relatively simple situation such Fig. S 2a (see SI section 4 for an example of formulating overlapping areas). In a focal square region, the number of overlapping area increases when the radius of detectable area increases, or bait interval decreases. If we incorporate trap efficacy, the results will imply that the bait interval needs to be decreased, which means that the number of overlapping area infinitely increases, which makes it impossible to formulate the observable ratio.

However, for a very limited situation we can analyse the outcomes of which trap efficacy is incorporated. Ending the analysis at a timing which $O(t)=1$ when $p=1$, there will be only one overlapping area per square region. The outcome is as following.

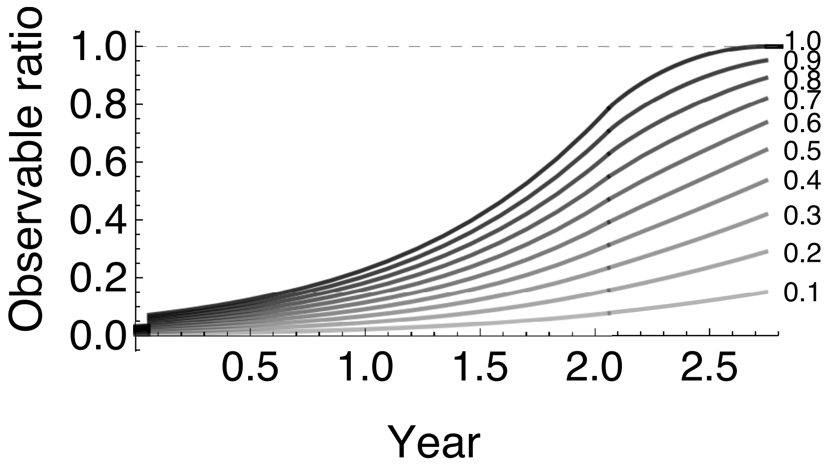

Fig. S 3 Observable ratio when incorporating trap efficacy, location of baits is not shifted.
Observable ratios are shown in curves. The numbers on the right side of the curve denotes the trap efficacy of which the observable ratio is calculated. Parameter set shown in Table 1 of the original paper is applied for calculation. The light gray dashed line denotes $O(t)=1$.

Fig. S 3 shows that the observable ratio increases depending on the trap efficacy. We previously showed that the observable ratio satisfies $O(t)<1$ if $p<1$, thus we can only conclude that "the observable ratio (and detection rate) increases depending on the trap efficacy, but will not reach one" if we incorporate such trap efficacy.

(2) To focus on the issue

Again, the purpose of this research is to show how wide the monitoring area has to be, and how intense should monitoring be on a spatio-temporal scale. The focus of this study is to investigate the

monitoring range and spatio-temporal pattern of traps required for theoretically thorough monitoring. From the above section it is shown that a theoretically thorough monitoring is not achievable if we incorporate trap efficacy. Furthermore, incorporating trap efficacy simply decreases the observable ratio, and no other helpful insights are obtained by the extension. In order to focus on the issue, it is necessary not to incorporate unhelpful assumption (an Occam's razor point of view).

2 The dispersal kernel

Dispersal kernel of nuptial flights in fire ants is poorly known, thus we simply assume a random walk type of dispersal. Then, the dispersal kernel will be a form of Gaussian distribution[2], and the dispersal kernel of the first generation $P_1(x,y)$ is

$$P_1(x,y)=\begin{cases}\dfrac{1}{2\pi\sigma^2}\exp\left(-\dfrac{x^2+y^2}{2\sigma^2}\right) & \left(\sqrt{x^2+y^2}\le 5\right)\\ 0 & \left(\sqrt{x^2+y^2}>5\right)\end{cases}. \quad \text{(S1)}$$

Here, $P_1(x,y)$ is a bivariate Gaussian distribution with mean μ satisfying $\mu=0$. $P_1(x,y)$ is zero when $\sqrt{x^2+y^2}>5$ since *S. invicta* females are assumed not to fly further than 5 km [3]. A distribution $P(x,y)$ is called a dispersal kernel when it satisfies $\iint P(x,y)\,\mathrm{d}x\,\mathrm{d}y=1$, and $P_1(x,y)$ may not seem to strictly satisfy this definition, however $\iint P_1(x,y)\mathrm{d}x\mathrm{d}y=0.9998\approx 1.0$, thus we will call $P_1(x,y)$ a dispersal kernel. The standard deviation σ depends on the average dispersal distance,

$$E_1(x,y)=\iint_{x,y}\left(\sqrt{x^2+y^2}\times P_1(x,y)\right)\mathrm{d}x\mathrm{d}y, \quad \text{(S2)}$$

which could be calculated based on laboratory experiment results[3] (see supplementary Box1). Solving the following equation,

$$1.62=E_1(x,y)=\iint_{x,y}\left(\sqrt{x^2+y^2}\times P_1(x,y)\right)\mathrm{d}x\mathrm{d}y, \quad \text{(S3)}$$

the estimated value of standard deviation is $\sigma\approx 1.2926$.

The dispersal kernel of the second generation $P_2(x,y)$ is

$$P_2(x,y)=\iint_{x_1,y_1} P_1(x_1,y_1)\times P_1(x-x_1,y-y_1)\mathrm{d}x_1\mathrm{d}y_1. \quad \text{(S4)}$$

Box 1

S. invicta females are known to fly for an average of approximately 45 minutes [3], with flight speed approximately 0.6 m/s when the temperature is 26 °C [3], which is the average of highest temperature of June 2017 in Kobe city, Japan [4].

Thus the average dispersal distance is $0.6\ \mathrm{m\cdot s^{-1}} \times 3600\ \mathrm{s\cdot hour^{-1}} \times 45/60\ \mathrm{hour} = 1620\ \mathrm{m} = 1.62\ \mathrm{km}$

3 Derivation of the detection rate

Consider the optimistic and pessimistic case. In the optimistic case, the source nest (first generation nest) is found instantly after it started producing queens, and second-generation alate queens have dispersed only for a short period (supplementary Fig. S 4a). In the pessimistic case, detection of the source and second-generation nest is delayed, such that the second-generation nest started producing queens and third-generation alate queens have dispersed for a short period (supplementary Fig. S 4b).

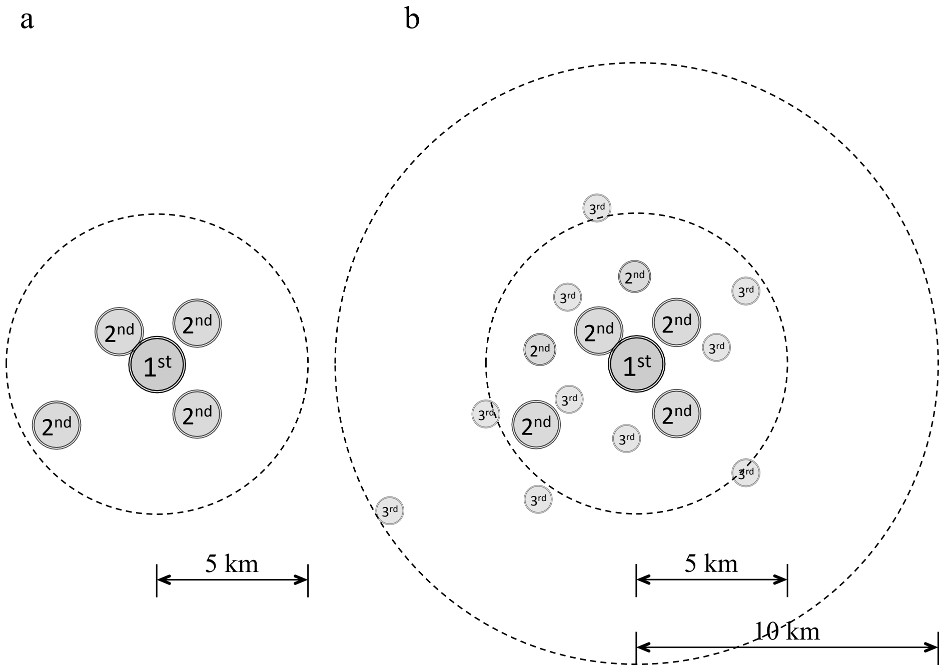

Fig. S 4 A possible distribution of the 1st, 2nd, and 3rd generation nests.

Circles with a number inside denote an area where fire ants from a nest would exist (with nests in the centre of the circle). The radius of a circle is $r(t)$ (see Table 1 in the original paper). The number scripted in the circle stands for the generation of the nest. (a) Optimistic case. (a) Pessimistic case.

Now let us assume that monitoring is conducted. Traps such as baits are set in lattice patterns. Then for an arbitrary nest, four closest baits from the centre of the nest could be selected, and there is a square with sides l_b (spatial interval of baits) (supplementary Fig. S 5a). In the square region, a nest would be detected if a bait was within $r(t)$ from the nest. However, a nest would also be detected if the nest was within $r(t)$ from the baits (supplementary Fig. S 5b). Thus, we shall call the area within $r(t)$ from the baits the *detectable area*. Whether an established nest is detected or not solely depends on whether the nest is within the detectable area or not, and the probability is given by a simple ratio, i.e. the width of the detectable area over the width of the square region. We will call this ratio the *observable ratio*.

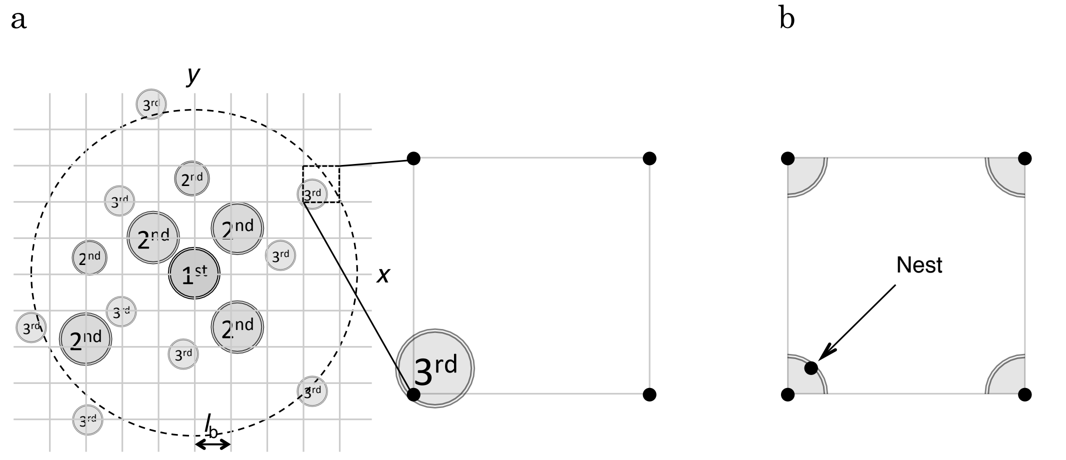

Fig. S 5 Definition of a detectable area.
(a) Bait traps are set in lattice patterns. A nest is inside a square region: sides are l_b, and there are baits on the corner. (b) Area within $r(t)$ from baits is the detectable area, which a nest would be detected if it was established inside.

The observable ratio obviously depends on $r(t)$, which differs among nests. However, the nest we shall consider is the one that is the most undetectable, since a monitoring strategy intense enough to detect the most undetectable nest would likely succeed in detecting every other nest. The most undetectable nest is the smallest nest, which is the youngest nest, which is the nest established at t_{1mat} in the optimistic case, and t_{2mat} in the pessimistic case (see Fig. 1 in the original paper). Here, the *detection rate* shall be defined as,

(Detection rate) =

∬{(Probability of the youngest nest establishing on coordinate (x,y)) (S5)

× (Probability of the youngest nest being detected after establishment)}dxdy,

while the first and second terms on the right hand side of equation (S5) are the dispersal kernel and the observable ratio, respectively. The observable ratio is time dependent. Thus, we obtain equation 1 in the original paper.

4 Detection rate when shifting bait locations

Assume that baits are located at $\left(x_i^t, y_j^t\right) = \left(i \times l_\mathrm{b}, j \times l_\mathrm{b}\right)$ ($i, j = 0, \pm1, \pm2, ..., \pm2 \times r_\mathrm{m}/l_\mathrm{b}$) during the first monitoring at time t (supplementary Fig. S 6a), where l_b is the spatial interval of baits and superscripts of x and y denote time. In the subsequent monitoring conducted at time interval t_int after the first, the baits are located at $\left(x_i^{t+t_\mathrm{int}}, y_j^{t+t_\mathrm{int}}\right) = \left(x_i^t + l_\mathrm{b}/2, y_j^t + l_\mathrm{b}/2\right)$ (supplementary Fig. S 6b).

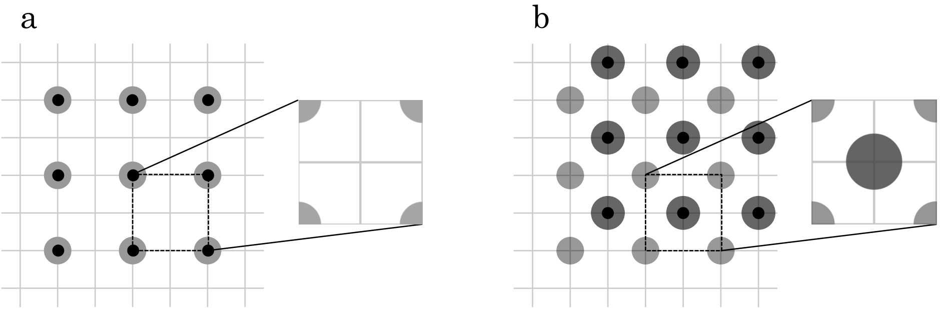

Fig. S 6 The overall detectable areas.

Black dots denote baits. Detectable areas in the first (light grey) and second (dark grey) monitorings are superimposed to show the overall detectable areas. (a) The first monitoring. (b) The second monitoring.

The detectable area will increase linearly while $r(t) + r(t + t_\mathrm{int}) < l_\mathrm{b}/\sqrt{2}$ (see supplementary Fig. S 7 a). When $r(t) + r(t + t_\mathrm{int}) \geq l_\mathrm{b}/\sqrt{2}$, the detectable areas at time t and $t + t_\mathrm{int}$ overlaps, and the total detectable area is not a simple sum of the detectable area at time t and $t + t_\mathrm{int}$. When defining angles θ and φ as shown in Fig. S 7b, the overlapping area in Fig. S 7b is given as equation (S6).

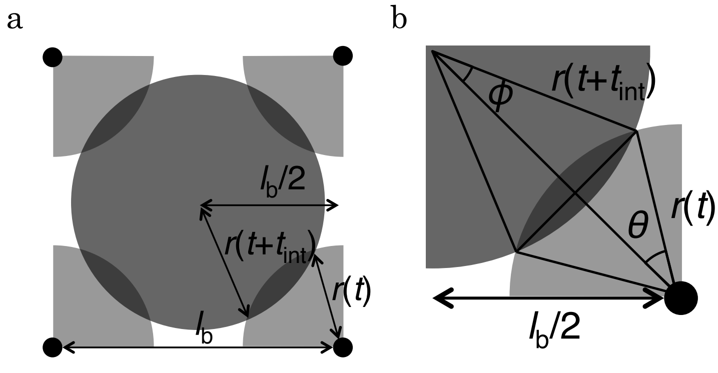

Fig. S 7 Definition of parameters.

Black dots denote baits. Detectable areas in the first (light grey) and second (dark grey) monitorings are superimposed to show the overall detectable areas. (a) The length of $r(t)$ and $r(t+t_\mathrm{int})$. (b) The angles.

$$
\begin{aligned}
&2\left\{\pi r(t)^2 \frac{\theta}{2\pi} - \frac{1}{2} r(t)\cos\theta \cdot r(t)\sin\theta + \pi r\left(t+t_{\mathrm{int}}\right)^2 \frac{\varphi}{2\pi} - \frac{1}{2} r\left(t+t_{\mathrm{int}}\right)\cos\varphi \cdot r\left(t+t_{\mathrm{int}}\right)\sin\varphi\right\} \\
&= r(t)^2\theta - r(t)^2\cos\theta\sin\theta + r\left(t+t_{\mathrm{int}}\right)^2\varphi - r\left(t+t_{\mathrm{int}}\right)^2\cos\varphi\sin\varphi \\
&= r(t)^2\left(\theta - \cos\theta\sin\theta\right) + r\left(t+t_{\mathrm{int}}\right)^2\left(\varphi - \cos\varphi\sin\varphi\right).
\end{aligned} \tag{S6}
$$

Here, $0 \le \theta, \varphi \le \pi/4$. Since parameters θ and φ are introduced for temporal use, it shall be replaced with $r(t)$ and $r\left(t+t_{\mathrm{int}}\right)$. Paying attention to the diagonal line in Fig. S 7b, they are given as equation (S7), (S8).

$$
r(t)\cos\theta + r\left(t+t_{\mathrm{int}}\right)\cos\varphi = \frac{l_{\mathrm{b}}}{\sqrt{2}} \tag{S7}
$$

$$
r(t)\sin\theta = r\left(t+t_{\mathrm{int}}\right)\sin\varphi \tag{S8}
$$

Since $0 \le \theta, \varphi \le \pi/4$, (S8) is equivalent to (S9).

$$
\cos\varphi = \sqrt{1 - \left(\frac{r(t)}{r\left(t+t_{\mathrm{int}}\right)}\sin\theta\right)^2} \tag{S9}
$$

Substituting equation (S9) to equation (S7) gives the relationship between θ, $r(t)$ and $r\left(t+t_{\mathrm{int}}\right)$.

$$
r(t)\cos\theta + \sqrt{r\left(t+t_{\mathrm{int}}\right)^2 - r(t)^2\sin^2\theta} = \frac{l_{\mathrm{b}}}{\sqrt{2}},
$$

$$
\therefore r\left(t+t_{\mathrm{int}}\right)^2 - r(t)^2\sin^2\theta = \frac{l_{\mathrm{b}}^2}{2} - \sqrt{2} l_{\mathrm{b}} r(t)\cos\theta + r(t)^2\cos^2\theta,
$$

$$
\therefore \sqrt{2} l_{\mathrm{b}} r(t)\cos\theta = \frac{l_{\mathrm{b}}^2}{2} + r(t)^2 - r\left(t+t_{\mathrm{int}}\right)^2,
$$

$$
\therefore \cos\theta = \frac{1}{\sqrt{2} l_{\mathrm{b}} r(t)}\left\{\frac{l_{\mathrm{b}}^2}{2} + r(t)^2 - r\left(t+t_{\mathrm{int}}\right)^2\right\},
$$

$$
\therefore \theta = \mathrm{Arc}\cos\left[\frac{1}{\sqrt{2} l_{\mathrm{b}} r(t)}\left\{\frac{l_{\mathrm{b}}^2}{2} + r(t)^2 - r\left(t+t_{\mathrm{int}}\right)^2\right\}\right]. \tag{S10}
$$

Similarly,

$$
\therefore \varphi = \mathrm{Arc}\cos\left[\frac{1}{\sqrt{2} l_{\mathrm{b}} r\left(t+t_{\mathrm{int}}\right)}\left\{\frac{l_{\mathrm{b}}^2}{2} + r\left(t+t_{\mathrm{int}}\right)^2 - r(t)^2\right\}\right]. \tag{S11}
$$

Similarly, formulate the detectable areas at $t = t + t_{\mathrm{int}}$ that will protrude from the square region (that is, when $\frac{l_{\mathrm{b}}}{2} \le r\left(t+t_{\mathrm{int}}\right)$ and $\theta < \frac{\pi}{4}$). Denote the angle as $\omega\left(= \frac{l_{\mathrm{b}}}{2r\left(t+t_{\mathrm{int}}\right)}\right)$ when formulating the

protruding areas. Then, when shifting bait locations, the observable ratio $O(t)$ could be given as following (however, I will leave θ, φ and ω unconverted for simplicity.).

$$\therefore O(t)=\begin{cases} \dfrac{\pi}{l_{\mathrm{b}}^2}\left(r(t)^2+r(t+t_{\mathrm{int}})^2\right) & if\left[r(t)+r(t+t_{\mathrm{int}})<\dfrac{l_{\mathrm{b}}}{\sqrt{2}}\right] \\ \dfrac{1}{l_{\mathrm{b}}^2}\begin{pmatrix} \pi\left\{r(t)^2+r(t+t_{\mathrm{int}})^2\right\}- \\ 4\begin{cases} r(t)^2(\theta-\cos\theta\sin\theta)+ \\ r(t+t_{\mathrm{int}})^2(\varphi-\cos\varphi\sin\varphi) \end{cases} \end{pmatrix} & if\left[r(t)+r(t+t_{\mathrm{int}})\geq\dfrac{l_{\mathrm{b}}}{\sqrt{2}}\cap r(t+t_{\mathrm{int}})<\dfrac{l_{\mathrm{b}}}{2}\right] \\ \dfrac{1}{l_{\mathrm{b}}^2}\begin{pmatrix} \pi\left\{r(t)^2+r(t+t_{\mathrm{int}})^2\right\}- \\ 4\begin{cases} r(t)^2(\theta-\cos\theta\sin\theta)+ \\ r(t+t_{\mathrm{int}})^2(\varphi-\cos\varphi\sin\varphi)+ \\ r(t+t_{\mathrm{int}})^2(\omega-\cos\omega\sin\omega) \end{cases} \end{pmatrix} & if\left[r(t+t_{\mathrm{int}})\geq\dfrac{l_{\mathrm{b}}}{2}\cap\theta<\dfrac{\pi}{4}\right] \\ 1 & if\left[\theta\geq\dfrac{\pi}{4}\right] \end{cases} \quad \text{(S12)}$$

The detection rate $D(t)$ could be calculated based on equation (S10), (S11), (S12) and (S13) as shown in the original paper.

$$D(t)=A_{\mathrm{m}}\times O(t). \quad \text{(S13)}$$

Where A_{m} is $A_{\mathrm{m}}=\iint_{-r_{\mathrm{m}}\leq x,y\leq r_{\mathrm{m}}} P(x,y)\mathrm{d}x\mathrm{d}y$, and r_{m} is the range of monitoring in x- and y- directions from the origin.

5 Importance of determining the source nest

Assume that multiple nests are found, both of which are considerably large and it is difficult to tell which nest is the source one. Then, they shall be carefully inspected to determine which nest is the source nest. The thoroughness of the monitoring will be lower than that shown in the original paper if a monitoring is conducted based on second- and third-generation nests. (See supplementary Fig. S 8)

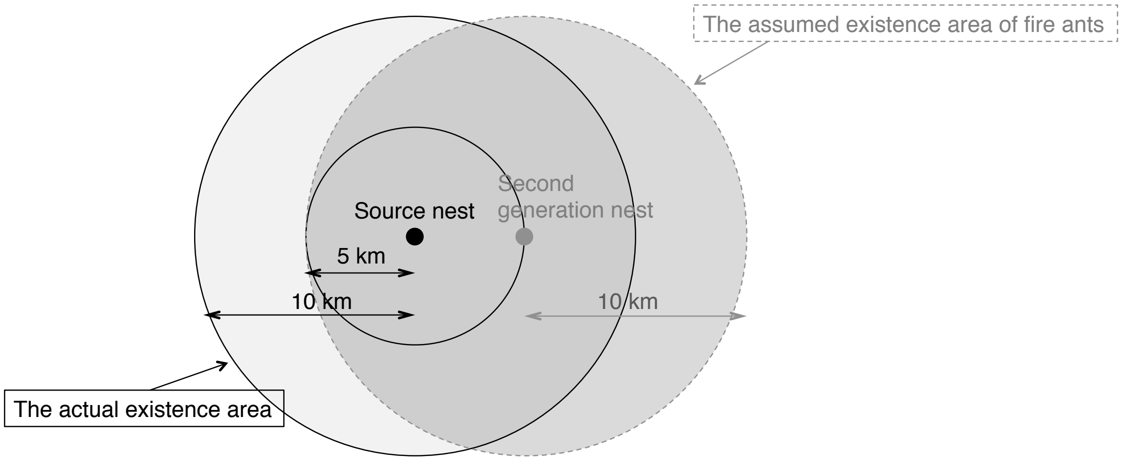

Fig. S 8 Difference between the assumed and actual existence area when monitoring is conducted based on second- and third-generation nests.
The actual existence area (light grey) and the assumed existence area (dark grey) of fire ants are superimposed. Monitoring based on the second-generation nest would lead to low thoroughness, resulting in low detection rate

6 Deriving minimum l_b from other parameters

When applying the model to other species, it would be convenient to have an equation to derive the minimum l_b (spatial interval of traps such as baits) required for the detection rate to be 1. Assume that the monitoring range covers the entire area the focal species may exist, i.e. $A_m = 1$ in equation (S13). Then, $O(t) = 1$ is required for $D(t) = 1$.

If $O(t) = 1$, the ratio of detectable area in a square region with sides l_b is 1 (see Fig. S 7a), which means that $\frac{l_b}{2r(t)} = \frac{1}{\sqrt{2}}$ if the location of baits is not shifted, and $\theta = \pi/4$ in Fig. S 7b if the location of baits is shifted. Thus if the location of baits is not shifted,

$$l_b = \sqrt{2}r(t). \quad \text{(S14)}$$

If the location of baits is shifted, substitute $\theta = \pi/4$ to equation (S10) to obtain

$$\frac{1}{\sqrt{2}} = \frac{1}{\sqrt{2}l_b r(t)} \left\{ \frac{l_b^2}{2} + r(t)^2 - r(t + t_{\text{int}})^2 \right\},$$

$$\therefore \frac{l_b^2}{2} - r(t)l_b + r(t)^2 - r(t + t_{\text{int}})^2 = 0,$$

$$\therefore l_b = r(t) \pm \sqrt{r(t)^2 - 4 \cdot \frac{1}{2} \cdot \left\{ r(t)^2 - r(t + t_{\text{int}})^2 \right\}},$$

thus considering the sign,

$$\therefore l_b = r(t) + \sqrt{2r(t + t_{\text{int}})^2 - r(t)^2}, \quad \text{(S15)}$$

where $r(t) = r_c(t) + r_s(t)$ and t_{int} is the temporal interval of monitoring. Equation (S15) could be

confirmed by increasing *t*. $r(t)$ is a logistic shape function and reaches its' maximum at around 7 years (see Fig.2 in the original paper). Thus if assuming $r(t) \approx r(t + t_{\mathrm{int}})$ when $t > 7$, then equation (S16) implies that $l_b = 2r(t)$, which is obviously correct (assume $r(t) = r(t + t_{\mathrm{int}}) = l_b/2$ in Fig. S 7a).

References

1 Bozdogan H. 1987. *Psychometrika* 52, 345-370.

2 Nathan R, Klein E, Robledo-Arnuncio JJ, and Eloy Revilla. 2012. Dispersal kernels: review in *dispersal ecology and evolution* (eds.) Clobert J, Baguette M, Benton TG and Bullock JM. Oxford University Press, 187-210.

3 Vogt JT, Appel AG, West MS, 2000. *J. Insect Physiol*. 46, 697-707.

4 Japan Meteorological Agency.
http://www.data.jma.go.jp/obd/stats/etrn/view/monthly_s3.php?prec_no=63&block_no=47770&year=&month=&day=&view=a2
